# Novel PSCA-Targeting Adapter Molecules for Late-Stage RevCAR-T Cell Therapy in Prostate Cancer

**DOI:** 10.3390/ijms27146407

**Published:** 2026-07-18

**Authors:** Claudia Arndt, Irene García de Andres, Ralf Bergmann, Nicola Mitwasi, Christin Neuber, Karla E. G. Soto, Nathalia Jones-Cifuentes, Alexandra von Jutrzenka-Trzebiatowski, Liliana R. Loureiro, Domokos Mathé, Michael Bachmann, Anja Feldmann

**Affiliations:** 1 Institute of Radiopharmaceutical Cancer Research, Helmholtz-Zentrum Dresden-Rossendorf (HZDR), 01328 Dresden, Germany; iregandres@gmail.com (I.G.d.A.); nicolam@miltenyi.com (N.M.); c.neuber@hzdr.de (C.N.); karlaelizabeth.gs@gmail.com (K.E.G.S.); n.jones-cifuentes@hzdr.de (N.J.-C.); a.von-jutrzenka-trzebiatowski@hzdr.de (A.v.J.-T.); lilianarloureiro@gmail.com (L.R.L.); mcbachmann@t-online.de (M.B.); a.feldmann@hzdr.de (A.F.); 2 Mildred Scheel Early Career Center, Faculty of Medicine Carl Gustav Carus, TUD Dresden University of Technology, 01307 Dresden, Germany; 3 Department of Biophysics and Radiation Biology—HUN-REN TKI, Semmelweis University, H-1094 Budapest, Hungary; rkbergmann@web.de (R.B.); domokos.mathe@hcemm.eu (D.M.); 4 Hungarian Centre of Excellence for Molecular Medicine, In Vivo Imaging Advanced Core Facility, H-6723 Szeged, Hungary; 5 School of Medicine, Department of Basic Sciences, Universidad Industrial de Santander, Bucaramanga 680002, Colombia; 6 German Cancer Consortium (DKTK), Partner Site Dresden, 01307 Dresden, Germany; 7 National Center for Tumor Diseases (NCT), NCT/UCC Dresden, A Partnership Between DKFZ, Faculty of Medicine and University Hospital Carl Gustav Carus, TUD Dresden University of Technology & Helmholtz-Zentrum Dresden-Rossendorf (HZDR), 01307 Dresden, Germany

**Keywords:** prostate cancer, CAR-T cells, RevCAR, PSCA, immunotherapy

## Abstract

Chimeric antigen receptor (CAR) therapies are emerging as promising strategies, particularly for metastatic castration-resistant prostate cancer (PCa), as they can act independently of the androgen receptor axis. Adapter CAR-T cell platforms, such as the RevCAR system, offer precise therapeutic control and tumor targeting via small, rapidly eliminated tumor-specific adapters. To enable more convenient late-stage RevCAR-T therapy in PCa patients, allowing for discontinuous reverse target module (RevTM) infusion, we developed novel, larger IgG4-based RevTMs targeting prostate stem cell antigen (PSCA) and benchmarked them against previously described smaller adapter formats. Within the RevCAR system, PSCA-IgG4 RevTMs effectively mediated PCa killing at low effector-to-target ratios and low RevTM concentrations in a strictly antigen-dependent manner. Oncolytic activity was accompanied by a rapid and pronounced release of proinflammatory cytokines across a broad RevTM concentration range, which is particularly advantageous for immunologically cold PCa. Finally, anti-tumor activity was confirmed in a short-term mouse model. Preliminary PET studies further indicate slow blood elimination and tumor-specific accumulation of novel IgG4-RevTMs. Together, these data position PSCA-IgG4 RevTMs as promising candidates for stepwise RevCAR-T treatment in PCa, in which short-lived scFv-RevTMs are initially used to ensure a rapid safety switch, followed by larger IgG4-RevTMs once the risk profile is known.

## 1. Introduction

Prostate cancer (PCa) still affects many men worldwide, accounting for 30% of cancers in men and being the second leading cause of cancer-related death [1]. The outcome for PCa patients has significantly improved over the last decade due to advanced imaging techniques [2,3,4], early diagnosis, and improved therapeutic options [5]. While curative treatment modalities exist for localized PCa, such as surgery, brachytherapy or external-beam radiation, the focus for more aggressive metastatic PCa (mPCa) is on multimodal treatments to improve long-term patient survival rates [5]. In mPCa, combinations typically involve standard chemo- or radiotherapies alongside androgen deprivation therapy or androgen receptor (AR) pathway inhibitors. However, the majority of patients with advanced PCa eventually progress to metastatic castration-resistant prostate cancer (mCRPC), a lethal disease with a poor prognosis and no curative treatment options [6], emphasizing the need for novel therapeutic interventions for this patient subgroup.

Targeted therapies are an emerging field in the management of mCRPC, encompassing diverse treatment modalities, such as recently approved enzyme poly(ADP-ribose) polymerase (PARP) inhibitors [7,8,9,10], radiopharmaceuticals [11,12], and immunotherapies [13]. Among immunotherapeutic approaches, chimeric antigen receptor (CAR)-T cells are a particularly powerful and promising approach, as evidenced by their remarkable efficacy in treating hematological diseases [14,15,16,17,18,19,20,21]. A multitude of ongoing clinical trials are currently investigating their therapeutic potential in PCa, addressing targets such as the prostate-specific membrane antigen (PSMA) [22,23], the prostate stem cell antigen (PSCA) [24,25,26], MUC-1 (NCT04483778), and STEAP-1 (NCT06236139), while both conventional and controllable CAR-T concepts are under investigation [27,28]. A recent Phase I trial reported promising biologic activity and a favorable safety profile of PSCA CAR-T cells in mCRPC patients [24]. This finding has led to the continuation of a Phase II trial (NCT05805371) aiming to explore the combination of PSCA CAR-T cells with or without radiotherapy to further boost anti-tumor effects. Another PSCA-targeting approach utilizes BPX-601 CAR-T cells that can be pharmacologically activated by the agent rimiducid via an inducible MyD88/CD40 switch, thereby offering a control mechanism in patients [26]. Nevertheless, the clinical study was halted due to the occurrence of severe immune-mediated adverse events, including grade 4 cytokine release syndrome. Overall, clinical findings underscore the potential but also the need to carefully balance efficacy and toxicity in CAR-T therapies.

**Figure 1:**
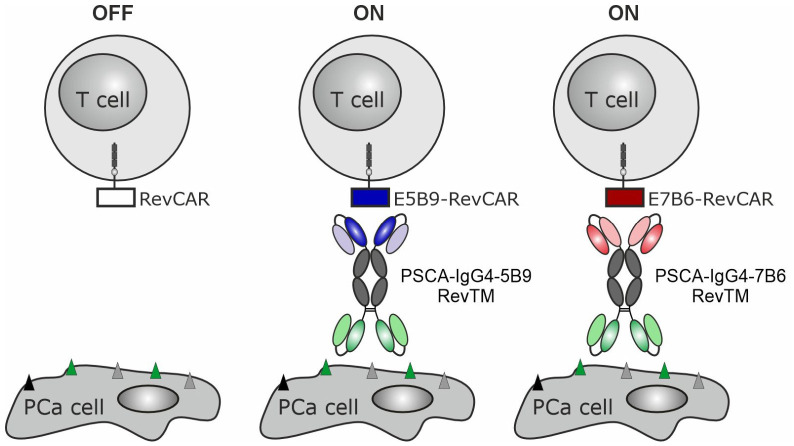
Schematic overview of novel PSCA-targeting RevCAR systems. The adapter CAR platform “RevCAR” consists of (i) T cells genetically engineered to overexpress either the E5B9- or the E7B6-RevCAR and (ii) bispecific adapters called reverse target modules (RevTMs). In steady-state RevCAR-T cells are switched “OFF” as they do not recognize any surface antigens (**left panel**). To exert cytotoxicity against tumor cells, the addition of cross-linking, tumor-specific RevTMs is required (“ON”, (**right panels**)). RevTMs with dual specificity for the prostate stem cell antigen (PSCA) and either the E5B9- (**middle panel**) or the E7B6-RevCAR (**right panel**) were designed to facilitate redirection of RevCAR-T cells to prostate cancer (PCa) cells.

An emerging alternative strategy that allows for improved controllability in patients involves the use of adapter CAR-T technologies, in which the activity of the CAR-T cells is precisely modulated through the administration of distinct tumor-specific adapter molecules [29,30,31,32,33]. This encompasses the peptide-based adapter CAR-T platforms UniCAR and RevCAR developed by our team [34,35], in which adapter CAR-T and adapter molecule interactions are based on short, non-immunogenic La peptides and their corresponding anti-La antibodies [36,37,38,39] (Figure 1).

**Figure 2:**
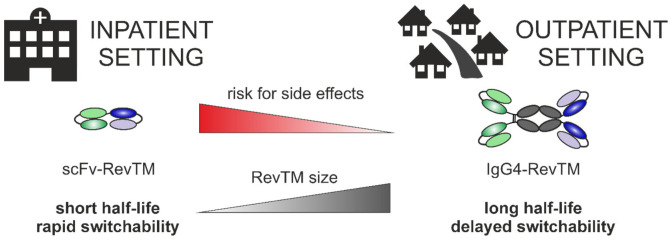
Envisioned stepwise RevCAR-T cell therapy with small and large adapters. Induction RevCAR-T therapy is performed in the hospital (inpatient setting) using small, short-lived scFv-RevTMs that are continuously infused, allowing for a rapid safety switch. Later, when the risk of side effects is low, the therapy can transition to an outpatient setting with discontinuous dosing of larger IgG4-RevTMs, which have an increased half-life but delayed switchability.

Due to their preclinical efficacy for targeting several hematological and solid cancers (e.g., [35,40,41,42]), both UniCAR and RevCAR systems have progressed to clinical trials (NCT04230265, NCT04633148, NCT05949125). Clinical findings from first-in-human UniCAR and RevCAR studies have demonstrated their biological safety, with only limited toxicities observed in patients with both acute myeloid leukemia (AML) and PCa [33,43,44,45]. Notably, both adapter platforms, when using antibody-based TMs, have achieved promising anti-tumor effects in AML [44,45]. UniCAR-T therapy resulted in overall response rates of 53% and 75% for minimal residual disease patients [44]. In contrast, the biological activity of UniCAR-T cells engaged via a small PSMA-L-derived adapter remained limited in PCa patients, which was most likely due to the rapid elimination of the PET-tracer-based TM. These outcomes underscore the necessity for further refinements of TMs, particularly in solid tumors. In this regard, optimizing and prolonging the pharmacokinetics of adapter molecules is a promising strategy to (i) sustain consistently high therapeutic levels in cancer tissues and (ii) maximize the clinical efficiency of adapter CAR-T in PCa, particularly in later stages of therapy. In addition, combining pharmacologically distinct RevTMs could facilitate a more convenient, stepwise RevCAR-T cell therapy without compromising safety control (Figure 2). In clinical practice, an initial treatment involving small, short-lived adapters could provide a rapid on/off control of RevCAR-T cells and ensure a high level of treatment safety. Subsequently, therapy could be transitioned to larger RevTMs once the safety risk profile can be more reliably assessed. In later stages of therapy, this would potentially eliminate the need for continuous adapter infusion and, consequently, for hospitalization.

In this study, we present the development and comparative, in-depth preclinical characterization of two novel IgG4-based adapter molecules with increased molecular size for use in our adapter CAR platform, “RevCAR,” to prospectively allow for longer-lasting therapeutic effects and outpatient maintenance therapy in mCRPC patients. We selected the PSCA as the target antigen due to its established role as a PCa-associated antigen. PSCA is known to be overexpressed in ~90% of PCa and has been found to positively correlate with advanced stage, metastatic disease and androgen-independence [46,47,48,49], making it particularly attractive for mCRPC interventions. New RevTM designs used the anti-PSCA domain from the monoclonal antibody (mAb) (MB1), for which comprehensive preclinical and even clinical phase 1 trial data are already available.

## 2. Results

### 2.1. Design of Novel PSCA-IgG4 RevTMs for the E5B9- and E7B6-RevCAR Systems

The RevCAR adapter technology is a two-component platform consisting of (i) T cells that have been genetically modified to express the RevCAR and (ii) bispecific adapters known as RevTMs (Figure 1). The RevCAR is a switchable, second-generation CAR that utilizes either the E5B9 or the E7B6 La peptide epitopes as the extracellular interaction domain, rendering it intrinsically inactive. The ability of E5B9- and E7B6-RevCAR T cells to elicit their cytolytic activity against cancer cells is strictly dependent on the presence of cross-linking RevTMs with dual specificity for the corresponding La peptide and a tumor target, like PSCA on PCa.

**Figure 3:**
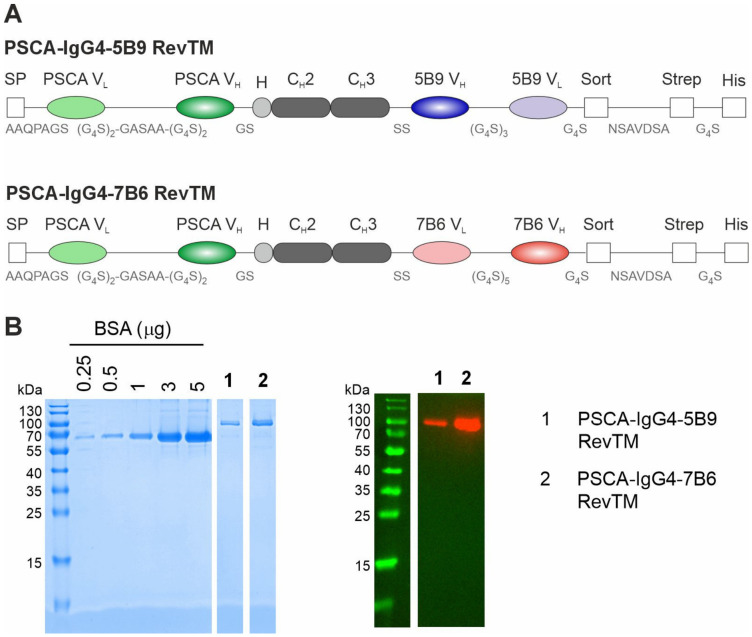
Molecular structure and purity of novel PSCA-IgG4 RevTMs. (**A**) Novel bispecific PSCA-IgG4 RevTMs were constructed by fusing the humanized anti-PSCA scFv (MB1) to the hinge (H) and Fc domains of human IgG4 as well as to the humanized anti-La scFvs (5B9 or 7B6), which recognize the E5B9 or E7B6 RevCAR epitope, respectively. The amino acid sequences of the connecting peptide linkers are provided below each RevTM. C_H_—constant domain of the IgG4 heavy chain, His—6x histidine-tag, Sort—sortase recognition site, SP—signal peptide, V_H_—variable domain of the heavy chain, V_L_—variable domain of the light chain. (**B**) PSCA-IgG4 RevTMs were purified via affinity chromatography using their C-terminal Strep-tag. Elution fractions were subsequently analyzed via SDS-PAGE. To determine the concentration of RevTMs using a predefined BSA standard and evaluate their purity, SDS gels were stained with Quick Coomassie^®^ Stain Solution ((**B**), left panel). Following Western blot-guided protein transfer onto a nitrocellulose membrane, immunochemical detection of RevTMs was performed using mouse anti-human IgG4-Fc HRP antibodies ((**B**), right panel). See Figure S1 for both the uncropped Coomassie-stained SDS gel and the Western blot.

To prospectively extend the therapeutic window of the previously described switchable RevCAR platform in PCa patients [35] and enable outpatient maintenance therapy, we herein introduce novel, larger IgG4-RevTMs. Specifically, the two novel PSCA-specific RevTMs were designed by fusing the humanized variable domain of the heavy (V_H_) and light chain (V_L_) of the anti-PSCA mAb (MB1) in V_L_-V_H_ orientation to the hinge and Fc domains (C_H_2-C_H_3) of human IgG4 molecules. To facilitate redirection of either E5B9- or E7B6-RevCAR T cells, the humanized anti-La single-chain fragment variables (scFvs) 5B9 (V_H_-V_L_ orientation) and 7B6 (V_L_-V_H_ orientation) were incorporated at the C-terminus, respectively (Figure 3A). The positioning of the anti-La scFv, the chosen V_H_-V_L_ orientation and the connecting linkers were kept the same as optimized for smaller, already established PSCA-5B9 and PSCA-7B6 RevTMs [35]. Both PSCA-IgG4 RevTMs were additionally equipped with an N-terminal signal peptide (SP, Igκ leader) and C-terminal purification tags (6xhistidine (His) and Strep). All functional elements of the novel IgG4-RevTMs were connected by short, flexible peptide linkers as specified in Figure 3A.

The novel PSCA-IgG4-5B9 and PSCA-IgG4-7B6 RevTMs were expressed in eukaryotic 3T3 production cell lines. The N-terminal murine Igk leader peptide mediates the secretion of RevTMs into cell culture supernatants. Using Strep-tag affinity chromatography, we successfully purified both RevTMs appearing as 83 kDa single polypeptide chains with high purity and quality in SDS-PAGE and Western blotting with subsequent immunochemical detection, as exemplified by one representative purification batch (Figure 3B). Overall, purification of RevTMs from cell culture supernatants yielded an average of 2.14 ± 0.84 mg/L PSCA-IgG4-5B9 RevTM and 3.79 ± 1.80 mg/L PSCA-IgG4-7B6 RevTM. Under physiological non-reducing conditions, the RevTMs form homodimers via disulfide bonds that covalently link the cysteine residues within the integrated hinge region (Figure 1). Thus, each RevTM represents a bispecific antibody with bivalently bound sites for the tumor target PSCA and the La peptide (E5B9 or E7B6).

### 2.2. Characterization of PCa Cell Line Models, E5B9- and E7B6-RevCAR T Cells

**Figure 4:**
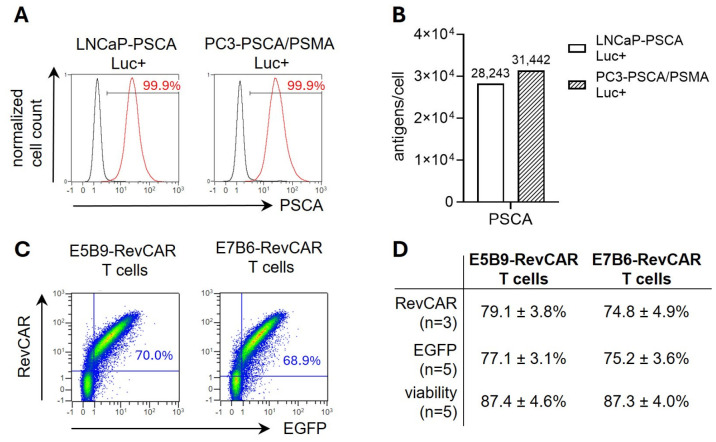
Characterization of PCa cell line models and RevCAR-T cells. (**A**) LNCaP-PSCA Luc+ and PC3-PSCA/PSMA Luc+ cells were stained with the anti-human PSCA mAb (MB1) and a goat anti-mouse IgG (H+L) Pacific Blue™, which served as the primary and secondary antibodies, respectively. Histograms show the percentage of positive cells (red curve) compared to the respective isotype controls (black curve). (**B**) PSCA surface expression was quantified using the QIFIKIT. The data show the number of PSCA molecules per cancer cell for one technical replicate. (**C**,**D**) To detect RevCAR surface expression, T cells were stained with anti-La mAb 5B9 or 7B6 for E5B9- or E7B6-RevCAR T cells, respectively. Binding was detected using Alexa Fluor^®^ 647 Goat anti-mouse IgG (minimal x-reactivity). To discriminate between live and dead cells, the cells were counterstained with propidium iodide. (**C**) RevCAR expression was plotted against expression of the co-translated EGFP marker protein. Dot plots show the percentage of RevCAR+EGFP+ double-positive cells for one representative donor. (**D**) The table provides an overview of the transduction efficacy and viability of E5B9- and E7B6-RevCAR T cells, generated from three to five distinct T cell donors.

To elucidate the functionality of the novel PSCA-IgG4 RevTMs in vitro, we used both AR-positive (LNCaP) and AR-negative (PC3) PCa cell line models. As both LNCaP and PC3 cells are known to downregulate PSCA expression under standard in vitro culture conditions [50], they were genetically modified to permanently and reliably overexpress PSCA [35] (Figure 4A). Quantitative flow cytometry revealed comparable PSCA surface expression to endogenously PSCA-expressing cell lines (e.g., LAPC-9, [51]) of approximately 28,000 and 31,000 molecules per cell for the LNCaP-PSCA Luc+ and PC3-PSCA/PSMA Luc+ cells, respectively (Figure 4B).

T cells were genetically modified to express either the E5B9- or E7B6-RevCAR using a one-week lentiviral transduction protocol. The efficacy of transduction was determined by measuring the level of co-expressed EGFP, which served as a surrogate marker protein. As demonstrated in Figure 4C, there is a strong correlation between EGFP and RevCAR surface expression, as detected via anti-La (5B9) or anti-La (7B6) mAbs for E5B9-RevCAR or E7B6-RevCAR T cells, respectively. As outlined in Figure 4D, the overall transduction efficacy was found to be high, with an average range of 75–79% observed for both RevCAR-T cell populations. The variability in transduction efficacy among different donors was low, and T cell viability remained high (~87%) (Figure 4D).

### 2.3. Novel PSCA-IgG4 RevTMs Bind with High Affinity Towards PSCA+ Tumor and RevCAR-T Cells

**Figure 5:**
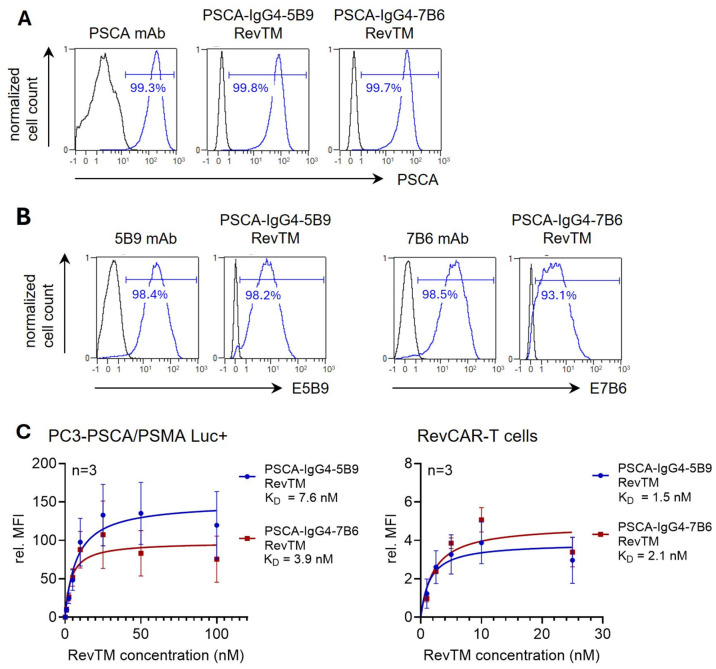
Binding properties of novel PSCA-IgG4 RevTMs. (**A**) PC3-PSCA/PSMA Luc+ tumor cells or (**B**) E5B9-RevCAR T cells or E7B6-RevCAR T cells were incubated with 25 nM of RevTMs. Subsequently, binding was detected via APC-conjugated anti-His Abs. (**A**,**B**) Histograms show the percentage of positive cells (blue curve) compared to the respective negative control staining (black curve). As a positive control, tumor and RevCAR-T cells were stained with anti-human PSCA mAb (MB1) and anti-human La mAb (5B9 or 7B6) as primary antibody, respectively. Their binding was detected with Alexa Fluor^®^ 647 Goat anti-mouse IgG (minimal x-reactivity) as secondary antibody. (**C**) To calculate the equilibrium dissociation constant (K_D_), PC3-PSCA/PSMA Luc+ or RevCAR-T cells were incubated with increasing concentrations of RevTMs, followed by staining with anti-His-APC Abs for detection. The RevTM concentrations were plotted against the relative median fluorescence intensity (rel. MFI) values. Graphs show summarized data from three technical replicates.

As a prerequisite for the functionality of the novel adapter CAR platforms, we first evaluated and compared the binding of novel PSCA-IgG4-5B9 and PSCA-IgG4-7B6 RevTMs towards PSCA and their corresponding RevCAR using flow cytometry. As shown in Figure 5A,B, both PSCA-IgG4 RevTMs specifically bind to PSCA-expressing PC3 tumor cells, and E5B9- or E7B6-RevCAR T cells. Binding towards PC3-PSCA/PSMA Luc+ cells occurred with K_D_ values of 7.6 nM and 3.9 nM for the PSCA-IgG4-5B9 and the PSCA-IgG4-7B6 RevTM, respectively (Figure 5C). Both PSCA-IgG4 RevTMs bound RevCAR-T cells with similar affinities of approximately 2 nM (Figure 5C).

### 2.4. PSCA-IgG4 RevTMs Specifically Redirect RevCAR-T Cells for Highly Efficient Killing of PSCA+ PCa Cells In Vitro

Functionality of the novel PSCA-directed E5B9- and E7B6-RevCAR systems was subsequently studied and compared using in vitro co-culture assays. For this purpose, RevCAR-T cells and tumor cells were incubated with or without the novel PSCA-IgG4 RevTMs. As demonstrated by a substantial CD69 upregulation across various RevTM concentrations (Figure S2A), both the PSCA-IgG4-5B9 and the PSCA-IgG4-7B6 RevTM were capable of mediating the activation of E5B9- and E7B6-RevCAR T cells upon cross-linking with PSCA-expressing tumor cells, respectively. In the presence of 10 nM or 0.7 nM of PSCA-IgG4-5B9 RevTM, CD69 expression was induced in 92.7% ± 3.2% and 91.6% ± 4.4% of E5B9-RevCAR T cells, respectively. Similarly, PSCA-IgG4-7B6 RevTM induced CD69 upregulation in 71.7% ± 8.3% (10 nM) and 71.8% ± 6.8% (0.7 nM) of E7B6-RevCAR T cells under the same conditions. The strength of CD69 stimulation decreased at lower concentrations for both TMs, reaching 77.8% ± 5.2% and 45.5% ± 4.1%, respectively, in the presence of 0.05 nM PSCA-IgG4-5B9 and PSCA-IgG4-7B6 RevTM. Overall, the extent of CD69 upregulation was found to be comparable across different T-cell donors and significant in comparison to the negative control without RevTM.

**Figure 6:**
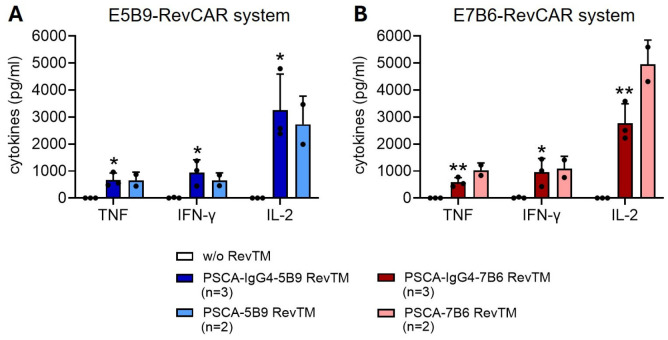
Cytokine profile of novel PSCA-IgG4 RevCAR systems. PC3-PSCA/PSMA Luc+ cells and (**A**) E5B9-RevCAR T cells or (**B**) E7B6-RevCAR T cells were incubated with or without 25 nM RevTM for 7 h at an E:T ratio of 5:1. Subsequently, cell-free supernatants were analyzed for TNF, IFN-γ and IL-2 using ELISA. The summarized data of three experiments using three or two independent T cell donors are shown. Statistical analysis was performed using multiple unpaired *t*-tests with Holm–Šídák correction for multiple comparisons; significance relative to w/o RevTM control is shown: ** *p* < 0.01, * *p* < 0.05. The data obtained for the small PSCA-scFv RevTMs were in line with their extensive previous evaluation [35]. As they served here only as reference controls and due to the small sample size of n = 2, they were excluded from statistical analysis.

**Figure 7:**
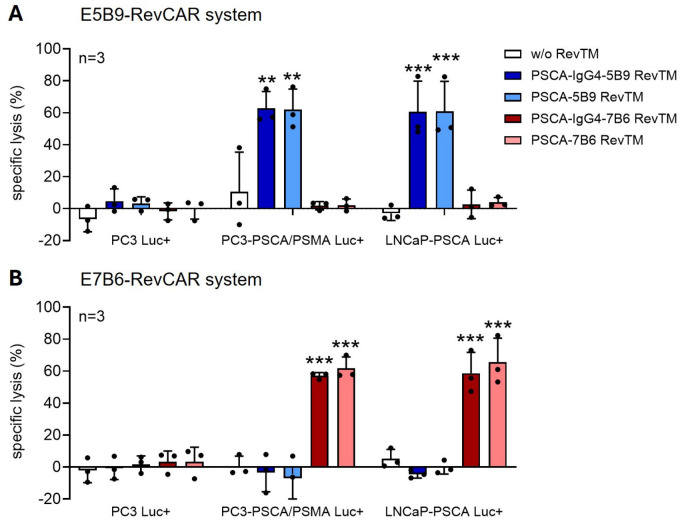
Comparison of the killing efficiency of PSCA-IgG4 RevTMs to PSCA-scFv RevTM against different cell lines. In 7 h luciferase-based cytotoxicity assays, PSCA^neg^ or PSCA^pos^ prostate cancer cells were co-cultured with (**A**) E5B9-RevCAR-T cells or (**B**) E7B6-RevCAR T cells in the absence or presence of 25 nM RevTM using an E:T ratio of 5:1. Mean specific lysis + SD for three independent experiments using three different T cell donors are shown (one-way ANOVA with Tukey’s multiple comparison test; significance relative to w/o RevTM control is shown: *** *p* < 0.001, ** *p* < 0.01).

Similar to the smaller scFv-based PSCA-E5B9 and PSCA-7B6 RevTMs described previously [35], PSCA-IgG4 RevTMs prompted RevCAR-T cells to secrete significant amounts of diverse T cell effector cytokines, including TNF, IFN-γ and IL-2, in the presence of PSCA+ tumor cells (Figure 6). This finally resulted in highly effective tumor cell killing within 7 h, as demonstrated for two different PSCA-expressing PCa cell lines (Figure 7).

A specific mode of action is an important prerequisite for the safe clinical use of the novel RevCAR systems. Thus, we explored whether bivalent RevTM binding to RevCAR-T cells alone would be sufficient to activate their effector functions in a target-independent manner. Additional co-culture assays of PSCA-negative target cells and/or RevCAR-T cells were performed in the absence or presence of RevTMs (Figure 7, Figures S2 and S3). Although at high concentrations, binding of PSCA-IgG4-5B9 RevTM alone or in the presence of PSCA-negative target cells induced CD69 upregulation on 40% of E5B9-RevCAR T cells (Figure S2B,C), it was insufficient to activate their effector mechanisms, as evidenced by the absence of T cell effector cytokines in co-cultures (Figure S3) and the inability to kill PSCA-negative tumor cells (Figure 7A). Similarly, E7B6-RevCAR T cells were unable to be activated by incubation with PSCA-IgG4-E7B6 RevTM alone in the absence of PSCA-expressing target cells, as indicated by low CD69 levels (Figure S2) and the absence of PSCA-negative target cell killing (Figure 7B). Moreover, co-incubation of RevCAR-T cells with non-matching PSCA-IgG4 RevTMs did not result in the killing of PSCA-positive tumor cells (Figure 7), which further underscores their specific mode of action.

**Figure 8:**
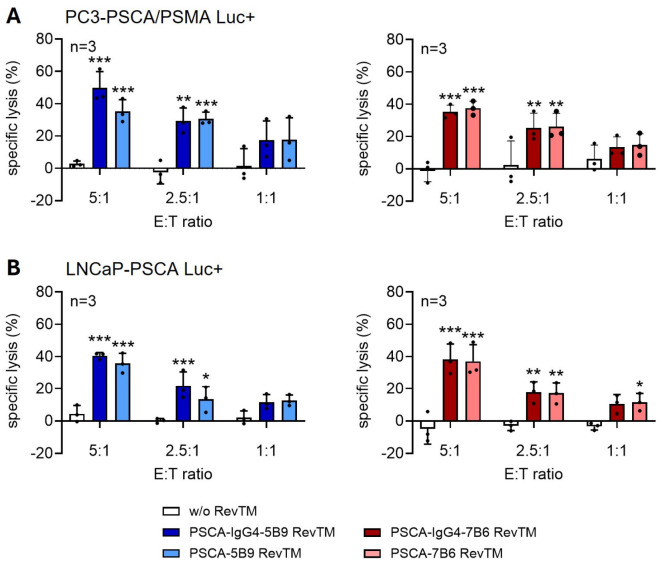
Killing efficiency of PSCA-IgG4 RevCAR systems at different E:T ratios. In 7 h luciferase-based cytotoxicity assays, (**A**) PC3-PSCA/PSMA Luc+ or (**B**) LNCaP-PSCA Luc+ cells were incubated with RevCAR-T cells with or without 25 nM RevTM at varying E:T ratios. Graphs show mean specific lysis + SD of three biological repeats using three different T cell donors (two-way ANOVA with Tukey’s multiple comparison test, significance relative to w/o RevTM control is shown: *** *p* < 0.001, ** *p* < 0.01, * *p* < 0.05).

To elucidate the activity limits of the novel RevCAR systems, we examined their killing efficiency under restrictive conditions using both AR-positive (LNCaP) and AR-negative (PC3) PCa cell lines. In particular, research was focused on lower effector-to-target cell (E:T) ratios and RevTM concentrations, as in patient settings, the availability of RevCAR-T cells and RevTMs might be limited. As summarized in Figure 8, PSCA-IgG4 RevTMs engaged their corresponding RevCAR-T cells after only 7 h for efficient lysis of both PSCA+ PC3 and LNCaP cell lines at low E:T ratios, although statistical significance was not yet reached at a 1:1 E:T ratio in all conditions. The oncolytic activity of novel PSCA-IgG4 RevTMs in combination with the respective RevCAR-T cells was thereby comparable to previously established scFv-based PSCA-5B9 and PSCA-7B6 RevTMs. In the absence of RevTMs, RevCAR-T cells were unable to lyse tumor cells, underpinning the RevTM-dependent mechanism of action of both RevCAR systems.

**Figure 9:**
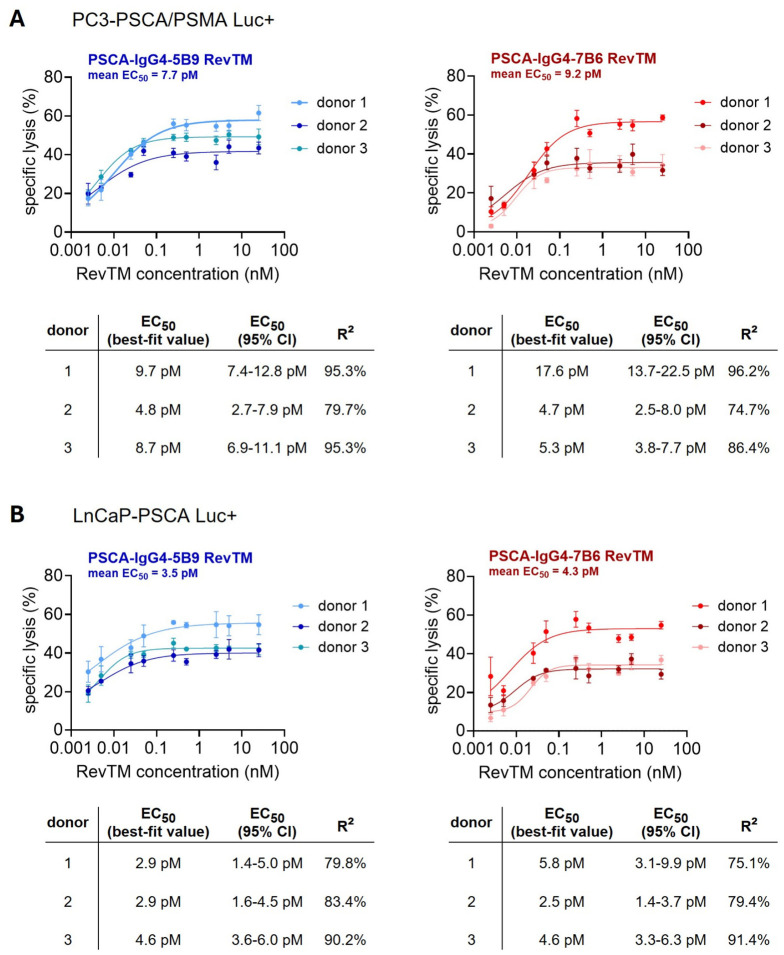
RevTM dose–response limits of the E5B9- and E7B6-RevCAR systems. In luciferase-based cytotoxicity assays, (**A**) PC3-PSCA/PSMA Luc+ or (**B**) LNCaP-PSCA Luc+ cells were incubated with RevCAR-T cells at an E:T ratio of 5:1 for 7 h. RevTMs were added at indicated concentrations. Graphs show mean specific lysis ± SD of three replicates for three individual T cell donors. EC_50_ calculations were performed by plotting the values on a nonlinear regression curve using GraphPad Prism software. Statistical information for each dose–response curve from an individual donor is provided below each graph. EC_50_—half-maximal effective concentration, CI—confidence interval, R^2^—coefficient of determination.

**Figure 10:**
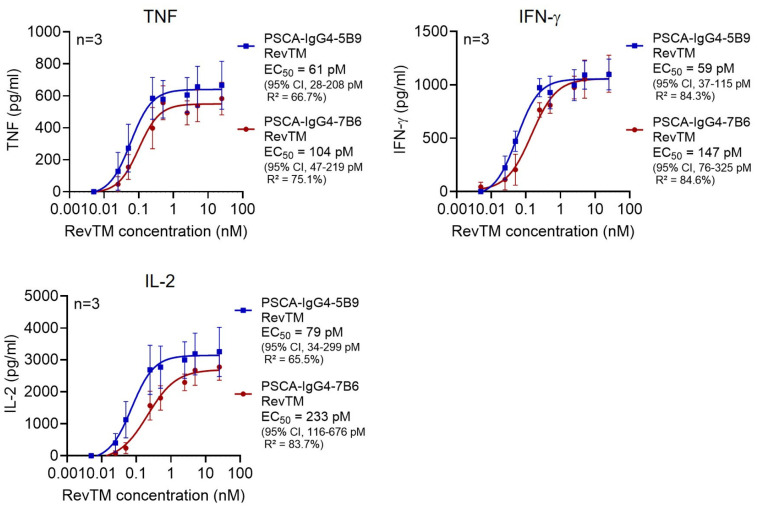
Cytokine profile of PSCA-IgG4 RevTM-redirected RevCAR-T cells. PC3-PSCA/PSMA Luc+ were incubated with RevCAR-T cells at an E:T ratio of 5:1. RevTMs were added at indicated concentrations. After 7 h, cell-free supernatants were harvested and analyzed for TNF, IFN-γ and IL-2 using sandwich ELISA. Graphs show mean cytokine concentration ± SEM of three biological repeats using three different T cell donors. EC_50_ calculations were performed by plotting the values on a nonlinear regression curve using GraphPad Prism software. EC_50_—half-maximal effective concentration, CI—confidence interval, R^2^—coefficient of determination.

To investigate the dose–response limits of the PSCA-IgG4 RevCAR platforms, we performed cytotoxicity assays with PSCA-positive PCa cell lines and RevCAR-T cells in the presence of decreasing RevTM concentrations. As shown in Figure 9, the half-maximal effective concentrations (EC_50_) of PSCA-IgG4-5B9 and PSCA-IgG4-7B6 RevTMs were in the low picomolar range (3–18 pM) for lysis of both AR-negative PC3-PSCA/PSMA Luc+ and AR-positive LNCaP-PSCA Luc+ cells. On average, the E5B9-RevCAR system demonstrated slightly higher efficiency at lower RevTM concentrations than the E7B6-RevCAR system. However, killing efficiency reached its plateau for both systems at a RevTM concentration of 0.25 nM. Interestingly, under the same conditions, the cytokine profile of RevTM-activated RevCAR-T cells exhibited lower sensitivity thresholds (Figure 10). Levels of the proinflammatory cytokines TNF and IFN-γ, as well as the growth-promoting cytokine IL-2, only peaked at a RevTM concentration of 2.5 nM. EC_50_ values for cytokine release were 8- to 25-times higher than EC_50_ values for tumor cell killing, with values ranging from 59 to 233 pM.

### 2.5. PSCA-IgG4 RevCAR Systems Are Effective in Short-Term Xenograft Mouse Models of PCa

**Figure 11:**
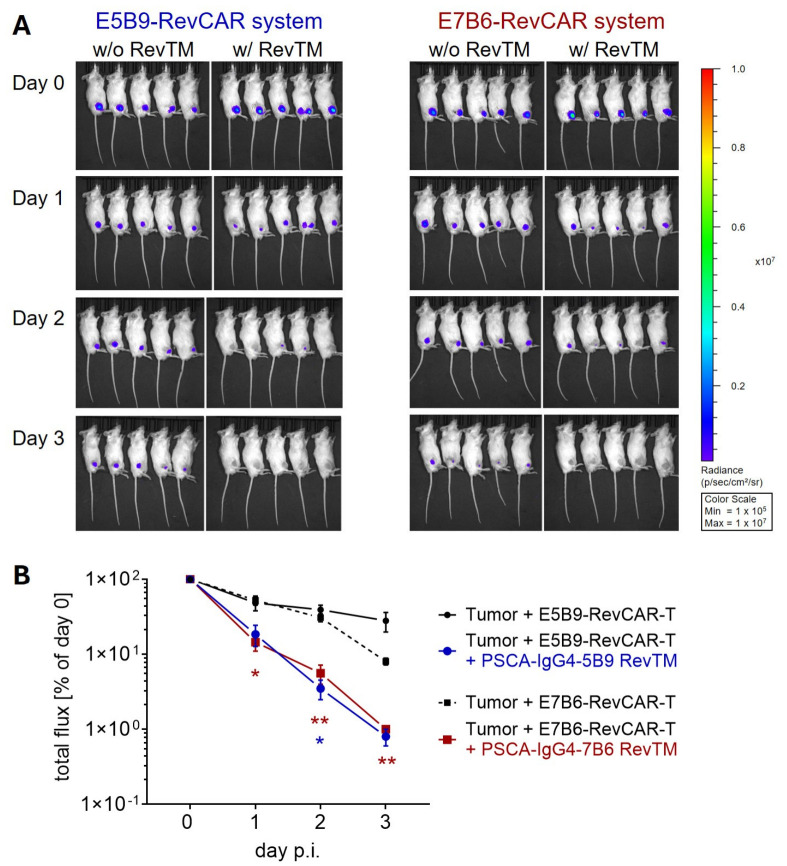
Therapeutic efficiency of novel PSCA-IgG4 systems in vivo. Immunodeficient NXG mice were subcutaneously injected into the right flank with cell mixtures of PC3-PSCA/PSMA Luc+ and RevCAR-T cells with (w/) or without (w/o) 100 pmol of the corresponding RevTM (n = 5). Mice were injected with luciferin solution to enable monitoring of tumor growth via optical imaging. (**A**) Luminescence images of each mouse are shown at 0, 1, 2, and 3 days after the start of the experiment. (**B**) The graph summarizes the bioluminescence signals as relative values (% of day 0). Results are plotted as mean ± SEM from five mice per group (two-way ANOVA with post hoc Šídák’s multiple comparisons test, compared to the control group w/o RevTM, ** *p* < 0.01, * *p* < 0.05).

To investigate the anti-tumor efficacy of the novel PSCA-IgG4 RevCAR systems in vivo, their functionality was investigated using a well-established subcutaneous co-injection mouse model. For this purpose, immunodeficient NXG mice were subcutaneously injected with one million PC3-PSCA/PSMA Luc+ cells and 500,000 RevCAR-T cells into the right flank. The treatment group additionally received 100 pmol of the PSCA-IgG4-5B9 RevTM or PSCA-IgG4-7B6 RevTM to specifically activate the cytolytic potential of E5B9- or E7B6-RevCAR T cells, respectively. Bioluminescence imaging was used to monitor tumor growth of luciferase-expressing PC3-PSCA/PSMA Luc+ tumor cells. As shown in Figure 11, bioluminescence signals of tumors in the treated group rapidly and significantly decreased within a few days, while tumor signals in the control group persisted. No differences were observed between the E5B9- and E7B6-RevCAR systems, proving a similar functionality also in vivo.

### 2.6. Biodistribution of Novel PSCA-IgG4 RevTMs

**Figure 12:**
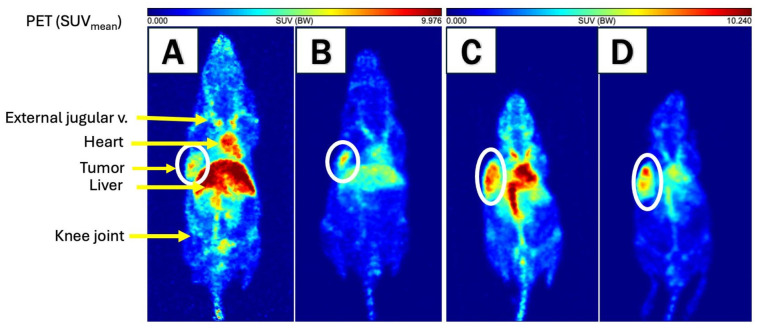
Maximum intensity projections of PET scans of PC3-PSCA bearing tumor mice. Mice were analyzed via PET imaging (**A**) 19 h, (**B**) 31 h, (**C**) 20 h, and (**D**) 32 h after a single intravenous injection of (**A**,**B**) [^64^Cu]Cu-NODAGA-PSCA-IgG4-5B9 RevTM or (**C**,**D**) [^64^Cu]Cu-NODAGA-PSCA-IgG4-7B6 RevTM. Radiolabeled RevTMs are clearly detectable in the tumors (white circles), the blood pool, liver and the bone marrow. The experiment was performed with one animal for each RevTM.

A first proof-of-concept positron emission tomography (PET) experiment was carried out to assess the biodistribution of the new PSCA-IgG4 TMs in vivo. After modification with NODAGA and radiolabeling with the diagnostic radionuclide Cu-64, two mice bearing PSCA+ PC3 tumors were injected with [^64^Cu]Cu-NODAGA-PSCA-IgG4-5B9 RevTM or [^64^Cu]Cu-NODAGA-PSCA-IgG4-7B6 RevTM, respectively. Due to the use of Cu-64 and its short half-life (t_1/2_ = 12.7 h), the evaluation of data via PET imaging was limited to two days. Figure 12 illustrates the observed biodistribution of both RevTMs in individual mice after 19 or 31 h, and after 20 or 32 h post-injection for the PSCA-IgG4-5B9 and the PSCA-IgG4-7B6 RevTM, respectively. Accumulation of the RevTMs was visible in the tumors, the blood pool and the liver. The accumulation in the liver most likely reflects the hepatic clearance [52] of the large PSCA-IgG4 RevTMs, but may also partly be explained by normal physiological copper metabolism [53]. The observed general distribution of RevTMs is comparable with the pattern previously described for the ^177^Lu-labeled anti-PSCA mAb (7F5) [54]. The activity concentration (SUVmean) for both RevTM (n = 1) from individual mice for tumor, heart (blood pool) and liver are summarized in Tables S1 and S2.

Although the PET data presented here are limited by the small sample size (n = 1 per construct), and thus, can only serve as an initial, qualitative proof-of-concept pilot experiment that requires further validation, they already show that both RevTMs were distributed within tumors, blood pool, liver and bone marrow for up to two days and thus indicate a trend toward prolonged retention and tumor accumulation.

## 3. Discussion

Metastatic PCa remains a significant therapeutic challenge. Despite the clinical benefits of integrating androgen deprivation therapy and novel AR-targeting agents (e.g., abiraterone, enzalutamide [55,56]) into first-line, docetaxel-based systemic therapy for advanced disease, patients ultimately progress and develop secondary resistance [57,58]. This highlights the urgent need for a paradigm shift toward alternative, more personalized treatment strategies that function independently of the AR axis. Beyond the currently available treatments, which include radioligand therapy (e.g., Lu-177–PSMA-617) [11], cabazitaxel [59], and PARP inhibitors [7,8,9,10], CAR-T cell immunotherapy represents an emerging research area in the treatment of recurrent mCRPC [60].

As potent and personalized targeted therapies, CAR-T cells hold great promise for overcoming the limitations of immunologically cold tumors, such as PCa [61], where other immunotherapies, e.g., checkpoint inhibitors, have proven ineffective [62]. Phase I clinical trials with CAR-T cells targeting diverse PCa-associated antigens have demonstrated promising signs of efficacy in patients with advanced PCa [24,60], but still do not achieve satisfactory results and are often associated with mild to severe adverse events, including fatal outcomes [23,60,63], which still pose significant safety concerns. Given the low physiological PSCA expression in healthy epithelial cells of, e.g., bladder, kidney, pancreas, stomach and large intestine [48,64,65], “on-target, off-tumor” toxicities against these tissues remain a major challenge for PSCA-targeted CAR-T therapies. In the first clinical PSCA-CAR T cell trials, side effects were limited to renal, urinary and gastrointestinal disorders [24,26]. The predominant tissue affected by "on-target, off-tumor" toxicity has been the bladder, with non-infectious cystitis being the sole dose-limiting toxicity, particularly in high-dose patient cohorts [24]. It is noteworthy that cystitis was likely exacerbated by standard lymphodepletion chemotherapy using cyclophosphamide [66], as a reduction in the dosage of cyclophosphamide has been shown to prevent high-grade cystitis [24]. However, to enhance the clinical translation of CAR-T cells in PCa and to maximize their efficacy while ensuring safety, it is imperative to manage and control therapy-related toxicities. Approaches such as CAR–γδ T cells and CAR-NK cells may be safer due to their limited lifespan, but inevitably result in the loss of costly engineered CAR products ([25], NCT04107142).

This is precisely where adapter CAR platforms, like the RevCAR system, may offer an advantage: they allow for the safe management of toxicity in CAR-T cell therapy through reversible on/off control. Specifically, RevCAR-T cell activity can be rapidly regulated by adjusting or stopping RevTM administration, thereby allowing immediate mitigation of potential side effects. This concept is supported by clinical data from phase I trials of the RevCAR-T [45] and the related UniCAR-T system in AML patients [33,43,44], where adverse effects were shown to be rapidly reversible after cessation of TM supply.

Until now, RevTMs targeting PCa have been based on scFvs as building blocks, resulting in relatively small bispecific adapters [35]. Their size of approximately 55 kDa favors rapid tissue penetration [67]. Being below the renal exclusion limit results in a short serum half-life and rapid renal clearance in vivo [67], as demonstrated previously for other bispecific antibodies or adapters [68,69]. This feature allows the RevCAR system to be quickly and temporarily switched off, enabling treatment pauses and rapid interventions when needed [33,43,44,45]. However, the short systemic persistence of small, rapidly eliminated adapters may limit effective dosing regimens in vivo, especially when sustained therapeutic exposure is necessary for successful treatment [70]. This seems particularly challenging for solid cancers, as evidenced by the initial results of the first-in-human study with the related UniCAR platform. While the UniCAR system elicited promising and sustained anti-tumor effects in AML [33,43,44], biological activity was evident but only limited in PCa patients (NCT04633148) when small tumor-specific adapters were applied via continuous infusion.

To overcome the aforementioned limitations and further boost the anti-tumor effects of adapter CAR-T cell therapy in PCa, the pharmacokinetic properties of adapter molecules were refined. Specifically, we here created novel, next-generation IgG4-based PSCA RevTMs to engage RevCAR-T cells for PCa killing. By incorporating an IgG4 backbone in the TM design, a strategy that has previously been successfully employed for other TMs (e.g., [69,71]), the molecules circulate longer in the blood and show tumor accumulation over 2 days (Figure 12). Mutations in the Fc region further provide greater molecule stability and minimize unwanted interactions with Fcγ receptor, thereby reducing the risk of off-target immune activation [72,73]. Notably, modifying the adapter molecule design and pharmacokinetics via IgG4-Fc integration preserves the switchable nature of the system while potentially enabling sustained therapeutic effects in patients without compromising the system’s safety or controllability. However, future longitudinal studies are required to verify prolonged RevCAR-T cell activity and sustained switchability of the system before clinical translation.

The novel PSCA-IgG4-5B9 and PSCA-IgG4-7B6 RevTMs investigated in this study have proven high-affinity binding in the low nanomolar range to both PSCA+ PCa cells and their corresponding RevCAR-T cells. Concurrent binding of RevTMs toward PSCA+ tumors and RevCAR-T cells has been shown to result in potent anti-tumor activity. Overall, the functional performance of the novel PSCA-IgG4 RevTMs was consistent across both RevCAR platforms. These results highlight the principal applicability of larger RevTMs within our adapter CAR systems and demonstrate that both RevCAR systems (E5B9 and E7B6) have high structural flexibility in terms of the selected RevTM format. Our functional comparisons have shown that larger IgG4-based RevTMs can be used equally well as smaller scFv-based RevTMs in the E5B9- and E7B6-RevCAR systems without impairing their overall functionality. This suggests that variations in immunological synapse geometry caused by different RevTM sizes do not impair RevCAR-T cell efficacy. This is in contrast to conventional CAR designs, where CAR-T cell performance can be negatively influenced by major size modifications in the extracellular CAR domains [74,75,76]. Importantly, novel PSCA-IgG4 RevTMs redirected RevCAR-T cells for effective killing of both AR-positive and AR-negative PCa cell lines within only seven hours of co-cultivation. The observed highly effective tumor killing, even at limiting concentrations of RevTM and at low E:T ratios, underscores the system’s potential to act efficiently in PCa tissues, where T-cell infiltration is known to be low [77]. For both the E5B9- and E7B6-RevCAR systems, RevTM-mediated oncolytic activity was accompanied by a rapid and significant release of proinflammatory and T cell-stimulating cytokines within a very short time across a wide range of RevTM concentrations. Notably, tumor cell killing occurred at lower RevTM concentrations than those required for maximum cytokine production. This suggests a clinically relevant therapeutic window. Administering lower PSCA-IgG4 RevTM doses may enable effective tumor cell killing while limiting cytokine release, thereby reducing the risk for systemic toxicities, such as cytokine release syndrome. This could be especially beneficial for long-term outpatient maintenance therapy in patients with low tumor burden. Conversely, higher levels of proinflammatory cytokines and thus higher PSCA-IgG4 RevTM doses may be desirable, particularly in PCa, an immunologically cold tumor [61,77], to overcome local immunosuppression and effectively stimulate CAR-T cell infiltration, effector function, survival, and potentially, the recruitment of other tumor-specific immune effectors. Overall, concentration-dependent differences in cytokine release and cytotoxicity thresholds provide a valuable pharmacologic tool for tailoring RevCAR treatment to individual patients’ needs, including safety requirements and tumor characteristics.

Finally, we successfully demonstrated the first in vivo proof-of-concept for the functionality of both RevCAR systems using a well-established co-injection PCa xenograft mouse model. While this setup validates the immediate anti-tumor activity of the novel PSCA-IgG4 RevCAR systems, it is limited to short-term observation. Despite this limitation, this animal model was selected for the following reasons: (i) to keep the burden for the experimental animals as low as possible, keeping in mind that (ii) data obtained with such animal models have proven sufficient for the regulatory approval of clinical phase 1 trials utilizing the UniCAR or RevCAR platforms. For future studies, the use of longitudinal tumor models may become relevant, focusing especially on the evaluation of sustained efficacy, safety, as well as RevCAR-T cell engraftment and persistence over extended time periods.

Given the bivalency of the novel PSCA-IgG4 RevTMs (Figure 1), precluding the risk of nonspecific RevCAR-T cell activation is crucial for clinical translation. Although the here presented bivalent RevTMs can potentially engage two RevCAR-T cells simultaneously or cross-link two RevCAR receptors on the same T cell, this study has proven that incubation of RevCAR-T cells with the PSCA-IgG4 RevTMs alone is insufficient to trigger relevant effector mechanisms of RevCAR-T cells, including cytokine release and the elimination of target-negative cell lines. This finding underscores the safety and the specific mode of action of the novel bivalent PSCA-IgG4 RevTMs.

As supported by our initial pilot PET imaging study, the novel PSCA-IgG4 TMs circulate in the blood pool, with visible tumor retention observed for at least up to two days. Due to the use of Cu-64 labeling, the observation window was limited. Thus, therapeutically relevant RevTM levels could potentially be sustained for even a longer period, which might extend tumoricidal RevCAR-T cell activity. Given the RevTM design and size, their general pharmacokinetics are expected to be similar to mAbs [54,67,78]. Thus, this new format might allow for a discontinuous dosing regimen, potentially eliminating the need for continuous infusions as required for small scFv-RevTMs. This alteration in the RevTM pharmacokinetic properties is promising for (i) intensifying anti-tumor effects in solid cancers, including PCa, and for (ii) later-stage therapy when the risk of side effects is low, and the ability to switch quickly is less important. Switching to a discontinuous RevTM application regimen could also improve patients’ quality of life and alleviate their treatment burden by reducing the necessity for prolonged hospitalization, which is typically required in the induction phase with rapidly switchable, small adapters. However, additional in vivo PET evaluations using larger cohorts and extended imaging timelines, as well as first-in-human trials, are necessary to fully characterize the pharmacokinetic profile of the novel IgG4-RevTMs, including in comparison to smaller scFv-RevTMs, and to define an optimal treatment regimen.

Based on the low accumulation of radiolabeled PSCA-IgG4 RevTMs in other organs and their specific tumor retention observed in our pilot PET study, these agents could be useful tools for imaging PCa in the future. The feasibility of TM-based PET imaging is supported by previous studies with ^68^Ga-labeled PSMA-L TM [40], which, similar to clinically used PSMA-11, enabled high-resolution visualization of tumor lesions in a PCa patient. In parallel, novel PSCA-IgG4 RevTMs may open new avenues for targeted radioimmunotherapy, an increasingly relevant strategy in the second-line treatment landscape for mCRPC [11,79,80]. In particular, longer-lived radiopharmaceuticals, like IgG4-RevTMs, could provide sustained tumor irradiation and improved therapeutic efficacy, especially in bulky or heterogeneous tumor lesions that may be less responsive to short-lived agents. However, full validation of the theranostic potential of novel PSCA-IgG4 RevTMs will require comprehensive in vivo PET and radioimmunotherapy studies in the future.

In summary, the novel PSCA-IgG4 RevTMs have demonstrated significant potential as immunotherapeutic adapters in the RevCAR platform, showing great potential to safely extend the treatment window of RevCAR-T cells for future PCa patient applications. Thereby, PSCA-IgG4 RevTMs could complement previously described adapters [35], which have a shorter serum half-life due to their small size. In the induction phase of therapy, when the potential adverse effects are not yet known, and the system requires greater adaptability, the employment of short-lived adapters could be a viable option. Subsequently, utilization of RevTMs with prolonged half-life could potentially sustain the therapeutic window, thereby facilitating more convenient outpatient management, particularly in cases of minimal residual and recurrent disease. Beyond the immunotherapeutic potential, future directions could use radiolabeled PSCA-IgG4 RevTMs to directly link the presented immunotherapeutic concept with cancer theranostics to further boost anti-tumor effects, particularly in mCRPC patients.

## 4. Materials and Methods

### 4.1. Cell Lines

PC3 and LNCaP cells were purchased from DSMZ (Leibniz Institute DSMZ-German Collection of Microorganisms and Cell Cultures, Braunschweig, Germany) and have not been further authenticated during the study period. Both PCa cell lines were modified to overexpress both PCa-specific target antigen(s) (PSMA and/or PSCA) and luciferase as previously described [35]. Cellular morphology and growth kinetics of PC3 and LNCaP cell lines were evaluated microscopically at every passage to confirm phenotypic consistency with standard parental cell lines. Furthermore, cells were regularly screened for PSCA, PSMA, and luciferase expression via flow cytometry. The murine fibroblast cell line 3T3 was used as a eukaryotic expression system. Genetic modification of 3T3 cells was achieved by lentiviral transduction to establish permanent RevTM production cell lines as reported before [81]. Tumor cell lines were cultured in RPMI 1640 w/o L-glutamine (Sigma Aldrich, Merck KGaA, Darmstadt, Germany) supplemented with 10% fetal calf serum, 1% penicillin/streptomycin, 1% non-essential amino acids, 1 mM Na-pyruvate and 2 mM glutamine (RPMI complete medium), while 3T3 production cells were kept in DMEM + GlutaMAX™-I media (Life Technologies Invitrogen, Thermo Fisher Scientific, Dreieich, Germany) supplemented with 10% fetal calf serum, 1% penicillin/streptomycin, and 1% non-essential amino acids (DMEM complete medium). All cells were maintained in a humidified atmosphere (37 °C and 5% CO_2_) and regularly screened for mycoplasma contamination via PCR.

### 4.2. Generation of RevCAR-T Cells

The ethical committee of the Medical Faculty Carl Gustav Carus, TUD Dresden University of Technology, Dresden, has given its approval for T cell studies (EK138042014). Human peripheral blood mononuclear cells (PBMCs) were isolated from buffy coats of healthy donors using density gradient centrifugation. Subsequent T cell isolation was performed using MACS technology employing the human Pan T cell Isolation Kit according to the manufacturer’s instructions (Miltenyi Biotec, Bergisch Gladbach, Germany). Lentiviral transduction of T cells with E5B9- or E7B6-RevCAR genes followed a previously published protocol [35]. In summary, 24 h after activation with T Cell TransAct^TM^ (Miltenyi Biotec), T cells were transduced on two consecutive days at a multiplicity of infection factor of 1. For expansion, RevCAR-T cells were finally seeded into G-Rex (Wilson Wolf Manufacturing LLC, Saint Paul, MN, USA) in TexMACS^TM^ medium (Miltenyi Biotec) supplemented with the T cell cytokines IL-2, IL-7 and IL-15. Prior to assays, RevCAR-T cells were maintained in cytokine-free RPMI complete media for 24 h.

### 4.3. Generation of PSCA-IgG4-RevTMs

The humanized scFvs from the murine anti-human PSCA (MB1) [81], anti-human La (5B9) and anti-human La (7B6) mAbs [36] were used as building blocks for the generation of novel PSCA-IgG4 RevTMs. Specifically, the PSCA-IgG4-5B9 RevTM was constructed by the connection of the humanized anti-PSCA scFv (MB1) to the hinge and Fc domains of human IgG4 and the humanized anti-La scFv (5B9) via flexible peptide linkers in one polypeptide chain. The PSCA-IgG4-7B6 RevTM was generated in a similar way, but using the anti-La scFv (7B6) to allow for E7B6-RevCAR T cell redirection. According to Schlothauer et al. [72], we introduced three amino acid mutations in the IgG4 hinge and C_H_2 domain of the novel PSCA-IgG4 RevTMs to stabilize RevTM dimerization via the hinge region and minimize its interaction with Fcγ receptors. All constructs further contain the murine Igκ leader peptide at the N-terminus, as well as two purification tags (Strep- and His-Tag) at the C-terminus. PSCA-IgG4 RevTM DNA sequences were generated in silico and finally cloned into the lentiviral expression vector p6NST50.

### 4.4. Expression and Purification of Novel PSCA-IgG4 RevTMs

To transduce and stably integrate DNA sequences of novel PSCA-IgG4 RevTMs into murine 3T3 expression cell lines, lentiviral particles were generated using newly cloned p6NST50_PSCA-IgG4 RevTM expression vectors according to a previously published lentiviral transduction protocol [81]. The resulting 3T3_PSCA-IgG4-5B9 and 3T3_PSCA-IgG4-7B6 production cell lines permanently produce and secrete the novel RevTMs in cell culture supernatants. The cell-free supernatant was then subjected to affinity chromatography using Strep-Tactin^®^XT 4Flow^®^ columns, following the manufacturer’s instructions (IBA Lifesciences GmbH, Göttingen, Germany). The eluates were dialyzed against 1xPBS overnight. Elution and wash fractions were quantitatively and qualitatively analyzed via discontinuous SDS-PAGE. Staining the SDS gels with Quick Coomassie^®^ Stain Solution (Serva, Heidelberg, Germany) allowed visualization of the protein bands. RevTM concentrations were determined using a predefined BSA standard on the same gel. The successful RevTM purification and enrichment in the eluates was confirmed by Western blotting and subsequent immunochemical detection using mouse anti-human IgG4-Fc HRP-conjugated antibodies (Southern Biotech, Birmingham, AL, USA) and Cytiva Amersham™ ECL™ Prime Western-Blot detection reagent (Fisher Scientific, Schwerte, Germany).

### 4.5. Evaluation of RevTM Binding, RevCAR and PSCA Expression via Flow Cytometry

Flow cytometry staining was performed with 1 × 10^5^ cells and 25 nM RevTM. RevTM binding was detected using anti-His APC Abs (1:100, Miltenyi Biotec). To determine the K_D_ values, target cells were stained with increasing concentrations of RevTMs ranging from 0.1 to 100 nM. The median fluorescence intensity (MFI) was plotted against the RevTM concentration. A one-site total binding curve fit was subsequently applied to calculate the K_D_ value using GraphPad Prism software, version 11.0.2 (92) (La Jolla, CA, USA).

PSCA expression on target cells was confirmed by staining with 10 μg/mL anti-human PSCA mAb (MB1) [81] and an Alexa Fluor^®^ 647 Goat anti-mouse IgG (minimal x-reactivity) Antibody (1:100; BioLegend, San Diego, CA, USA) as the primary and secondary antibodies, respectively. PSCA levels on the cell surface of the PCa cell lines LNCaP-PSCA Luc+ and PC3-PSCA/PSMA Luc+ were quantified by flow cytometry using the Quantitative Analysis Kit (QIFIKIT^®^, Agilent/Dako, Santa Clara, CA, USA), following the manufacturer’s instructions. Briefly, 2 × 10^5^ cells were incubated with 15 µg/mL of anti-human PSCA mAb (MB1) or 15 µg/mL of the respective mouse IgG1, κ isotype control (BD Biosciences, Heidelberg, Germany). Binding was subsequently detected using a 1:50 dilution of goat anti-mouse IgG (H+L) cross-adsorbed secondary antibody, Pacific Blue™ (Thermo Fisher Scientific).

EGFP expression served as a surrogate marker for the successful transduction of T cells with RevCAR constructs. Their surface expression was verified by indirect immunofluorescent staining using 20 μg/mL anti-La mAbs (5B9 or 7B6) [36] and Alexa Fluor^®^ 647 Goat anti-mouse IgG (minimal x-reactivity) Antibody (1:100; BioLegend).

Shortly before each flow cytometric analysis, the stained cells were incubated with Invitrogen^TM^ propidium iodide (1:1000 in 1xPBS; Life Technologies GmbH, Darmstadt, Germany) to discriminate between live and dead cells. Immunofluorescently stained cells were acquired using a MACSQuant 10 flow cytometer (Miltenyi Biotec). The data were analyzed using MACSQuantify software, version 2.13.3 (Miltenyi Biotec).

### 4.6. Activation Assays

RevCAR-T cells were cultured in 96-well U-bottom plates in the presence or absence of PSCA-positive or PSCA-negative target cells. RevTMs were added at concentrations of 0.05, 0.7, or 10 nM. After incubating for 24 h, co-cultures were centrifuged for 5 min at 360× *g*, and cell-free supernatant was removed. Then, RevCAR-T cells from each triplicate condition were pooled, washed with 1xPBS, and stained for 15 min with anti-human CD69-APC antibody (Miltenyi Biotec) to analyze their activation status. PI counterstaining was used for live–dead cell discrimination. Data were acquired on a MACSQuant 10 flow cytometer (Miltenyi Biotec). The percentage of CD69+ RevCAR-T cells was determined by analyzing the PI-negative, EGFP-positive cell population.

### 4.7. Enzyme-Linked Immunosorbent Assay (ELISA)

Tumor cells and RevCAR-T cells were cultured with or without RevTMs at indicated concentrations and an E:T ratio of 5:1. Cell-free supernatant was harvested, centrifuged (5 min, 360× *g*), and analyzed for the T-cell cytokines TNF, IFN-γ, and IL-2 using the following ELISA kits in combination with the BD OptEIA Reagent Set B: Human TNF ELISA Set, Human IFN-Gamma ELISA Set, and Human IL-2 ELISA Set (BD Biosciences). All reagents were used according to the manufacturer’s instructions. The EC_50_ RevTM concentrations were determined by plotting measured cytokine concentration against tested RevTM concentrations on a nonlinear regression curve using GraphPad Prism software (La Jolla, CA, USA).

### 4.8. Luciferase-Based Cytotoxicity Assays

In vitro functionality of novel PSCA-IgG4 RevTM was studied using luciferase-based cytotoxicity assays, following a previously described method [82]. Specifically, 5 × 10^3^ luciferase-expressing tumor cells were incubated with RevCAR-T cells at the indicated E:T ratios and RevTM concentrations. After 7 h, specific lysis was determined using the ONE-Glo™ Luciferase Reagent (Promega GmbH, Mannheim, Germany). The readout was performed on an Infinite^®^ M200 Pro microplate reader (Tecan Germany GmbH, Crailsheim, Germany).

### 4.9. In Vivo Studies

All procedures involving experimental mice were conducted in accordance with the ARRIVE guidelines, the European Communities Council Directive (86/609/EEC) guidelines, and the German Animal Welfare Regulations. The experimental mice were housed under standard conditions, with a 12 h day/night cycle, and were fed autoclaved water and food ad libitum.

Approval for evaluation of in vivo functionality via bioluminescence imaging was granted by the local Ethical Committee for Animal Experiments at the Landesdirektion Sachsen (DD24.1-5131/449/67). Experiments were conducted with 10-week-old, male NXG mice (NOD-Prkdc^scid^-IL2rg^Tm1^/Rj, Janvier Labs, Le Genest-Saint-Isle, France) following a previously published protocol [35]. Briefly, animals were subcutaneously injected into the right flank with cell mixtures of 1 × 10^6^ PC3-PSCA/PSMA Luc+ and 0.5 × 10^6^ RevCAR-T cells with or without 100 pmol of the corresponding RevTM (n = 5). Mice were randomly assigned to the treatment or control groups (fixed randomization). To monitor tumor growth over time, mice were anesthetized (10% desflurane in 0.5 L min^−1^ oxygen/air 1:4 (*v*/*v*)), injected with 200 µL D-Luciferin (15 µg/mL in PBS, PerkinElmer, Rodgau, Germany) and subsequently analyzed via optical imaging using the IVIS Spectrum CT system and corresponding Living Image^®^ 4.7.4 (64-bit) software (PerkinElmer) on days 0, 1, 2, and 3. Mice were monitored every two to three days during the experiment. The animals had to be sacrificed if the tumor size was ≥15 mm, if there was weight loss of >20%, tumor ulceration, or if the animals appeared to be suffering, in accordance with animal welfare regulations.

PET studies were conducted following the approval of the Animal Care and Use Committee of the IEM and the Semmelweis University (PE/EA/293-7/2021).

^64^Cu-labeled RevTMs were washed three times by spin filtration (10 min, 3500× *g*, 7 °C) through an Amicon^®^ Ultra centrifugal filter (Merck KGaA, Darmstadt, Germany) with a 100 kDa molecular weight cut-off in PBS containing 0.1 mM EDTA to remove free Cu-64 and equilibrate the solution to PBS for injection. To study the biodistribution of novel PSCA-IgG4 RevTMs via PET imaging, male Crl:NMRI-*Foxn1nu* mice (Charles River Laboratories, Wilmington, MA, USA) received a single subcutaneous injection of 1 × 10^6^ PC3-PSCA/PSMA-Luc+ cells in their right shoulders. The biodistribution of ^64^Cu-labeled RevTMs in the mice was visualized by PET imaging, as previously described [71], using a NanoScan^®^ PET/MRI 3T (Mediso, Budapest, Hungary). The study was conducted on two mice. Mice received a single intravenous injection of 8.36 MBq [^64^Cu]Cu-NODAGA-PSCA-IgG4-5B9 RevTM, and 9.13 MBq of [^64^Cu]Cu-NODAGA-PSCA-IgG4-7B6 RevTM, with body weights of 23.69 g and 20.36 g, respectively. PET measurements were performed at two different time points post RevTM injection: at 19 h or 31 h, and 20 h or 32 h for the PSCA-IgG4-5B9 or PSCA-IgG4-7B6 RevTM, respectively. The reconstructed images were analyzed using InterView^®^ Fusion 3.01.016.0000 (Mediso, Budapest, Hungary) and Rover (ABX GmbH, Radeberg, Germany). Image-derived regions of interest (ROI) were expressed as mean SUV (g/mL) ± SD, where n is the number of voxels. The small number of animals did not allow for further statistical evaluation.

### 4.10. Statistics

Statistical analyses were conducted using GraphPad Prism software (GraphPad Software Inc., Boston, MA, USA). In vitro experiments were performed with a sample size of n = 3 independent T cell donors (biological replicates), if not otherwise stated. Each biological replicate was measured in three technical replicates to ensure technical precision. To test whether the data followed a normal Gaussian distribution, the D’Agostino-Pearson omnibus test (α = 0.05) was used. For individual cytokine comparisons (Figure 6), the sample sizes per variable were too small to perform formal normality testing, so the data were assumed to be normally distributed based on visual inspection. Statistically significant differences between the control group (w/o RevTM) and the two treatment groups (PSCA-IgG4 RevTM and PSCA-scFv RevTM) were determined using one-way or two-way ordinary ANOVA followed by Tukey’s multiple comparisons test, as specified in the figure legends. When statistical analysis included only one control and one treatment group, multiple unpaired *t*-tests with Holm–Šidák correction for multiple comparisons were applied.

Non-linear regression curves for the determination of EC_50_ values were calculated according to the three-parameter logistic model, with the bottom set to the value corresponding to the “w/o TM” condition.

In our animal study, a Two-way repeated measures ANOVA with a post hoc Šidák’s multiple comparisons test was used to assess statistical differences between the treatment (PSCA-IgG4 RevTM) and respective control group (w/o RevTM).

The applied statistical tests and number of biological repeats are also specified in the respective figure captions. Differences were considered statistically significant at a p-value of less than 0.05 (*** *p* < 0.001, ** *p* < 0.01, * *p* < 0.05) and a 95% confidence interval (CI).

## Data Availability

The original contributions presented in this study are included in the article/Supplementary Material. Further inquiries can be directed to the corresponding author.

## Supplementary Materials

The following supporting information can be downloaded at: https://www.mdpi.com/article/10.3390/ijms27146407/s1.

## Abbreviations

The following abbreviations are used in this manuscript:

AML	acute myeloid leukemia
AR	androgen receptor
CAR	chimeric antigen receptor
EC50	half-maximal effective concentration
ELISA	enzyme-linked immunosorbent assays
E:T	effector-to-target
His	6x histidine
mAb	monoclonal antibody
mCRPC	metastatic castration-resistant prostate cancer
mPCa	metastatic prostate cancer
MFI	median fluorescence intensity
PARP	poly(ADP-ribose) polymerase
PBMC	peripheral mononuclear cells
PCa	prostate cancer
PET	positron emission tomography
PSCA	prostate stem cell antigen
PSMA	prostate-specific membrane antigen
RevCAR	reverse chimeric antigen receptor
RevTM	reverse target module
V_H_	variable domain of the heavy chain
V_L_	variable domain of the light chain
