# Supplementary material for: Novel PSCA-Targeting Adapter Molecules for Late-Stage RevCAR-T Cell Therapy in Prostate Cancer
Source: Int J Mol Sci. 2026 Jul 18;27(14):6407. doi: 10.3390/ijms27146407 (PMC13410071; doi:10.3390/ijms27146407)
Supplement: Supplementary file 1 [file ijms-27-06407-s001.zip › ijms-4387507-supplementary.pdf]

Supplementary Material

Novel PSCA-Targeting Adapter Molecules for Late-Stage RevCAR-T Cell Therapy in Prostate Cancer

Claudia Arndt, Irene García de Andres, Ralf Bergmann, Nicola Mitwasi, Christin Neuber, Karla E. G. Soto, Nathalia Jones-Cifuentes, Alexandra von Jutrzenka-Trzebiatowski, Liliana R. Loureiro, Domokos Mathé, Michael Bachmann and Anja Feldmann

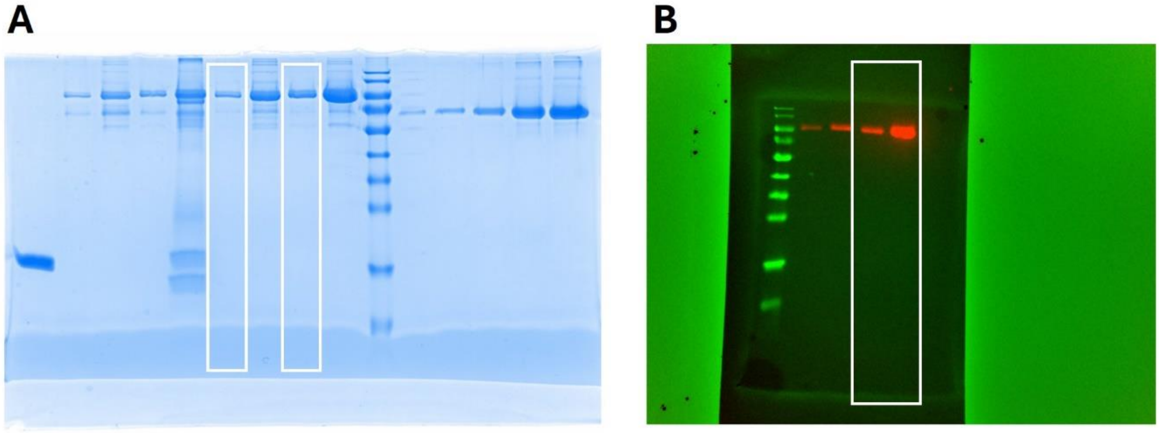

Figure S1. Uncropped **(A)** Coomassie-stained SDS gel and **(B)** Western blot images. The lanes in the white boxes represent the PSCA-IgG4-5B9 RevTM (left) and the PSCA-IgG4-7B6 RevTM (right) shown in Figure 3B.

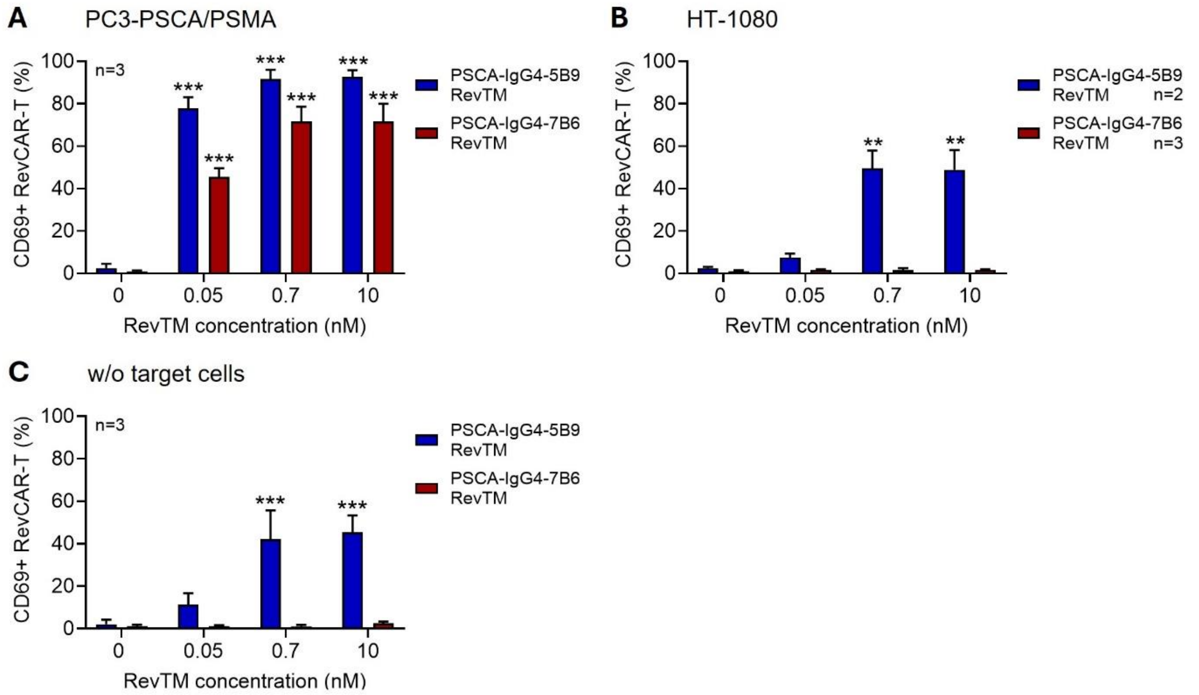

Figure S2. Upregulation of early activation marker CD69 via novel PSCA-IgG4 RevTM. RevCAR-T cells were incubated with **(A)** PC3-PSCA/PSMA cells, **(B)** PSCA-negative HT-1080 cells or **(C)** without target cells in the presence of their corresponding RevTM at an E:T ratio of 5:1. After 24 hours, RevCAR-T cells were stained with anti-human CD69-APC Abs. Graphs show mean percentage of CD69+ RevCAR-T cells + SD from two or three experiments using two or three independent T cell donors (one-way ANOVA with Dunnett´s multiple comparison test; significance relative to w/o RevTM control is shown: *** $p < 0.001$, ** $p < 0.01$, ** $p < 0.05$).

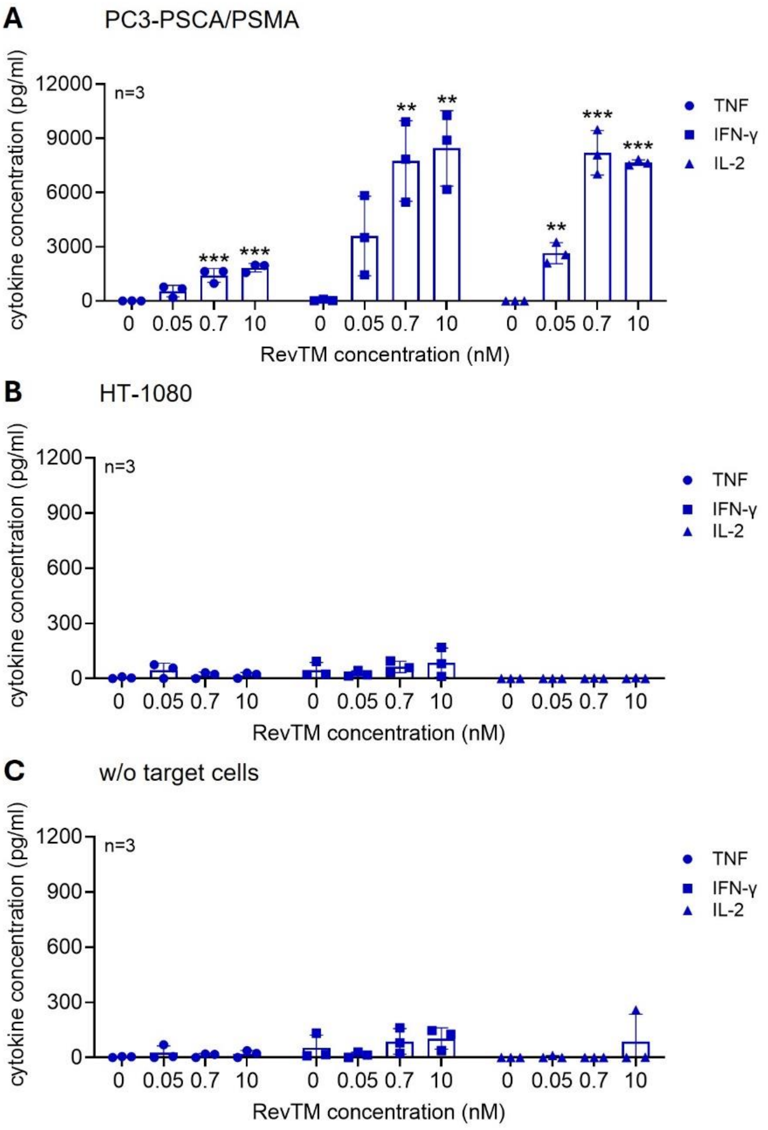

Figure S3. Cytokine profile of PSCA-IgG4-5B9 RevTM. E5B9-RevCAR T cells were incubated with **(A)** PC3-PSCA/PSMA cells, **(B)** PSCA-negative HT-1080 cells or **(C)** without target cells in the presence of the PSCA-IgG4-5B9 RevTM at an E:T ratio of 5:1. After 24 hours, cell-free supernatants were analyzed for TNF, IFN-γ and IL-2 using ELISA. Summarized data of three experiments using three independent T cell donors are shown (one-way ANOVA with Dunnett´s multiple comparison test; significance relative to w/o RevTM control is shown: *** $p < 0.001$, ** $p < 0.01$, ** $p < 0.05$).

Table S1. [^{64}Cu]Cu-NODAGA-PSCA-IgG4-5B9 RevTM distribution calculated from the PET images after 19 and 31 hours. The region of interest (ROI) values are presented as mean ± SD from n voxels in the ROI.

	Time (h)	SUVmean	SD	n
**Tumor**	19	4.64	0.81	2,294
	31	3.77	0.91	1,177
**Heart**	19	6.09	1.24	2,220
	31	2.70	0.61	1,120
**Liver**	19	7.67	1.63	9,215
	31	3.65	0.81	10,689

Table S2. [^{64}Cu]Cu-NODAGA-PSCA-IgG4-7B6 RevTM distribution calculated from the PET images after 20 and 32 hours. The region of interest (ROI) values are presented as mean ± SD from n voxels in the ROI.

	Time (h)	SUVmean	SD	n
**Tumor**	20	5.50	1.18	6517
	32	3.98	1.03	4495
**Heart**	20	8.00	1.65	1909
	32	4.42	0.83	1995
**Liver**	20	5.63	0.78	503
	32	3.48	0.29	503
